# Time Irreversibility and Criticality in the Motility of a Flagellate Microorganism

**DOI:** 10.1103/PhysRevLett.121.058103

**Published:** 2018-08-03

**Authors:** Kirsty Y. Wan, Raymond E. Goldstein

**Affiliations:** 1 Department of Applied Mathematics and Theoretical Physics, Centre for Mathematical Sciences, University of Cambridge, Cambridge CB3 0WA, United Kingdom; 2 Living Systems Institute, University of Exeter, Exeter EX4 4QD, United Kingdom

## Abstract

Active living organisms exhibit behavioral variability, partitioning between fast and slow dynamics. Such variability may be key to generating rapid responses in a heterogeneous, unpredictable environment wherein cellular activity effects continual exchanges of energy fluxes. We demonstrate a novel, noninvasive strategy for revealing nonequilibrium control of swimming—specifically, in an octoflagellate microalga. These organisms exhibit surprising features of flagellar excitability and mechanosensitivity, which characterize a novel, time-irreversible “run-stop-shock” motility comprising forward *runs*, knee-jerk *shocks* with dramatic beat reversal, and long *stops* during which cells are quiescent yet continue to exhibit submicron flagellar vibrations. Entropy production, associated with flux cycles arising in a reaction graph representation of the gait-switching dynamics, provides a direct measure of detailed balance violation in this primitive alga.

In his *De Incessu Animalium*, Aristotle had described the walk of a horse [1]: “[T]he back legs move diagonally in relation to the front legs, for after the right fore leg animals move the left hind leg, and then the left foreleg, and finally the right hind leg.” Since Aristotle, the control of locomotion in most animals is now understood to be enabled by central pattern generators [2], yet despite lacking a nervous system, certain primitive microeukaryotes can also actuate micro-scale analogues of limbs called cilia and flagella to produce swimming gaits akin to the trot and gallop of quadrupeds [3]. These microorganisms are not restricted to a single gait but rather are capable of multiple: classic examples include the run and tumble of *E. coli* [4], the run-reverse-flick motility of *V. alginolyticus* [5], and the numerous escape gaits of the ciliate *P. tetraaurelia* [6]. Such heterogeneity of movement (in terms of speed or directionality) is conserved across multiple species and is crucial for effecting rapid responses within a dynamic and unpredictable environment [7].

To avoid the perpetual tendency toward disorder, living organisms take in free energy by consuming adenosine triphosphate, rendering the intracellular milieu a hub of activity whose nonequilibrium nature is revealed when the thermodynamic fluctuation-dissipation theorem (FDT) is violated during microrheological responses to weak external forcing [8]. At more macroscopic scales, microscopic breaking of detailed balance may be disguised or even partially restored. Inference of departure from equilibrium is further hampered by the absence of a generalized FDT, prompting the development of novel, noninvasive strategies rooted in the identification of phase-space currents [9–11]. Here, we show how violation of detailed balance may be detected at the level of a free-living organism.

We consider motility control in the flagellate marine alga *Pyramimonas octopus* [12] (Fig. 1), which belongs to a fascinating group of unicellulars bearing 2^*k*^ flagella. These exhibit a delicate interplay between passive (fluid mechanical) and active intracellular control of flagella [3]. Cells are oblong or rectangular in aspect (Fig. 1), with length 17.05 ± 1.74 *μ*m and width 9.05 ± 1.23 *μ*m. Three gaits were consistently identified—the minimum number required for emergence of cycles or flux loops in a discrete representation. A forward *run* gait *O*(1) s in duration requiring synchronous, breaststroke coordination of diametrically opposed flagella pairs is interrupted by abrupt (< 100 ms) episodes involving dramatic changes in flagella beating, hereafter termed *shocks*. The third is a long-lived, *O*(10) s, *stop* gait in which there is no cell body movement but yet minute flagellar oscillations.

We explore each of the gaits in turn [Fig. 2(a)]. Compared to bacteria, the larger size of these algae facilitates visualization [details in the Supplemental Material (SM) [13]], allowing us to associate changes in flagellar beating unambiguously with gait transitions, and thence with reorientation of swimming trajectories. When swimming freely, cells spin about their long axis, with a significant 3D component. However, by restricting ourselves to individuals traversing the focal plane, we observe the flagella distinctly. In a stereotypical sequence stop⇀shock⇀run, a cell initiates spontaneously a run from rest via a shock [Figs. 2(b) and 2(c)]. Defining the instantaneous alignment $D=\hat{v}\cdot {\hat{e}}_{R}$ between the swimming direction $\hat{v}$ and the cell body axis **ê**_*R*_, the pullerlike run (*D* = 1) may be distinguished from the pusherlike shock (*D* = −1), during which flagella are transiently thrown in front of the cell [Fig. 2(a)]. Concomitantly, the beat pattern transitions from a bilateral ciliary to an undulatory flagellar beat [18]. Averaged over ten cells, the translational speed rises rapidly from zero to 1712 ± 392 *μ*m/s, but relaxation to a mean run speed of 428 ± 64 *μ*m/s takes 50 ms. To separate flagellar motion from body orientation, we track two dynamically morphing regions *𝒜* and *ℬ* that are delineated by image intensity: an inner one for the cell body and an outer one bounding the flagella (see the SM [13]). The length *λ*(*t*) = ‖ Σ_**x**∈*ℬ*\*𝒜*_**x***/* |ℬ\*𝒜*| − Σ_**x**∈*𝒜*_**x***/*|*𝒜*|‖ measures the physical separation between the centers of mass of the flagella and the cell body, where ‖ · ‖ is the Euclidean norm and | · | the number of pixels enclosed. Next, we present gait switching in speed-shape (*v, λ*) space. For both a single transition [Fig. 2(d)] and an average over multiple [Fig. 2(e)], the stop state exhibited minimal shape fluctuations, while transitions from stops to runs via shocks appear as loops with two distinct branches: an excitatory portion involving rapid changes in *speed*, and a refractory period associated with changes in *shape* [Fig. 2(e)].

To estimate transition probabilities between gaits, we implement a continuous-time Markov model, using instantaneous speed *v* to automate a three-state gait discretization from digitized tracks [Fig. 3(a)]. The state variable *X*(*t*) takes the values {0 = stop; 1 = run; 2 = shock}. States are positive recurrent and the process is irreducible. The Markov assumption is well supported empirically by measuring waiting time distributions between states. The transition probability matrix *P*(*t*), with *p*_*ij*_ = *P*[*X*(*t* + *τ*) = *j*|*X*(*τ*) = *i*] = *P*[*X*(*t*) = *j*|*X*(0) = *i*], satisfies *dP / dt* = *P*(*t*)*Q*, where *Q* = {*q*_*ij*_} is the infinitesimal rate matrix, with *q*_*ij*_ = lim_Δ*t*→0_ *𝒫*[*X*(Δ*t*) = *j*|*X*(0) = *i*]/Δ*t* (*i* ≠ *j*), and *q*_*ii*_ = −Σ_*j*≠*i*_*q*_*ij*_. We estimated *Q* (see the SM [13]) from *O*(10^4^) s of cumulative recordings (individual track durations of 0.5–80 s), totaling 1377 distinct pairwise transitions obtained from 233 cells:

(1) $$\begin{array}{l} \begin{array}{cccc} & \;\;\;\;\;\;\;\;\;\;\;\;\;\;\;\;\;\;\;\;\;\;\;\;\;\;\text{stop}\; & \;\;\;\;\;\;\;\;\text{run}\; & \;\;\;\;\;\;\;\;\;\;\;\;\;\;\;\text{shock}\; \end{array}\\ Q=\begin{array}{c} \;\text{stop}\;\\ \;\text{run}\;\\ \;\text{shock}\; \end{array}\left[\begin{array}{l} -0.132\;\;\;\;\;\text{0}.008\;\;\;\;\;\;\;\;\;\;\;\;\;\;0.124\\ \;0.281\;\;\;\;\;-1.330\;\;\;\;\;\;\;\;\;\;\;\;\;1.049\\ \;0\;\;\;\;\;\;\;\;\;\;\;\;\;\;\;\;\text{19}.\text{77}\;\;\;\;\;\;\;\;\;\;\;-19.77 \end{array}\right]({\text{s}}^{-1}). \end{array}$$

The zero-eigenvalue left eigenvector of *Q* dominates *P*(*t*) for large values of *t*, producing a unique equilibrium distribution *P*(∞) = *π*(stop, run, shock) = (0.6666, 0.3126, 0.0208). This is in good agreement with an alternative estimate of relative dwell times (68.6%, 30.8%, 0.6%) obtained by histogram binning of speeds [Fig. 3(b)]. The latter uses a larger data set which additionally includes tracks with no transitions and subjective cutoffs (stop, 0 ≤ *v* ≤ 40; run, 40 ≤ *v* ≤ 500; shock, *v* ≥ 500 *μ*m/s). Drawing an analogy with chemical reaction rates, our continuous-time process admits an embedded Markov chain {*k*_*ij*_*, i* ≠ *j*} with entries *k*_*ii*_ = 0 (no self-transitions), *k*_*ij*_ = *q*_*ij*_*/*Σ_*l*≠*i*_*q*_*il*_, Σ_*j*_*k*_*ij*_ = 1, ∀ *i*, representing the probability of *i* → *j* transitions conditioned on discrete “jump times” {*T*_*n*_}, such that *T*_*n*+1_ = inf{*t* ≥ *T*_*n*_|*X*(*t*) ≠ *X*(*T*_*n*_)}. Here, *k*_01_ = 0.0582, *k*_02_ = 0.9418, *k*_10_ = 0.2112, *k*_12_ = 0.7888, *k*_20_ = 0, and *k*_21_ = 1.0000 [Fig. 3(c)]. Sojourn times *T*_*n*+1_ − *T*_*n*_ are exponentially distributed with rates −*q*_*ii*_, from which we compute expected waiting times for stop, 7.60 ± 0.75 s; run, 0.75 ± 0.03 s; and shock, 0.05 ± 0.002 s (uncertainties are standard errors).

The process is clearly irreversible, as run⇌shock transitions occur readily, yet the direct reaction shock⇀stop is never observed; the Kolmogorov criterion for detailed balance is violated: *k*_01_*k*_12_*k*_20_(= 0) ≠ *k*_02_*k*_21_*k*_10_(= 0.187). We define an entropy production rate $\dot{S}$,

(2) $$\dot{S}\mathrm{:=}\frac{1}{2}\sum_{i\neq j}J_{ij}A_{ij}\geq 0,$$

from fluxes ***J***_*ij*_ = *π*_*i*_*k*_*ij*_ − *π*_*j*_*k*_*ji*_ and conjugate forces *A*_*ij*_ = ln (*π*_*j*_*k*_*ij*_*/π*_*i*_*k*_*ji*_) to characterize the difference between forward andtime-reversed entropies. $\dot{𝒮}$ can also be interpreted as the sum of the time derivative of the internal Gibbs entropy and an additional term due to nonequilibrium driving [19]. In steady state, ${\dot{P}}_{i}(t)=\sum_{j}\left(p_{j}k_{ji}-p_{i}k_{ij}\right)=0$ for *p*_*i*_ = *π*_*i*_, so $\dot{S}$ reduces to

(3) $$\dot{S}\frac{1}{2}\sum_{i\neq j}\left(\pi_{i}k_{ij}-\pi_{j}k_{ji}\right)\ln\left(\frac{k_{ij}}{k_{iji}}\right).$$

For apparently “irreversible” reactions that are not observed over the course of the experiment, we avoid *k*_*ji*_ = 0 by taking *k*_*ji*_ = (*π*_*j*_***T***_max_)^−1^, where ***T***_max_ = 78.17 s is the maximum single-track duration, to obtain $\dot{𝒮}=0.249$. Thus, $\dot{𝒮}$ *quantifies* the lack of detailed balance in the nonequilibrium steady state, which, as reported elsewhere [20], depends on environmental conditions, emphasizing the need to account for nonequilibrium effects in theoretical models. Such effects are not at all obvious at the mesoscale: while breaking of detailed balance occurs in two-state chemotaxis motility strategies when gait-transition rates vary in space [21], or alternatively in the presence of spatially asymmetric obstructions (e.g., funnel ratchets) [22], bacteria run and tumble with spatially constant parameters can, nonetheless, be mapped to free Brownian diffusion, which satisfies detailed balance.

Figures 3(d)–3(f) show the morphology of the three primary sequences: run⇀shock⇀run, stop⇀shock⇀run, and run⇀stop. Typical of photosynthetic unicells [23], forward swimming is quasihelical with superimposed self-rotation. Tracks comprise low-curvature runs and sharp turns due to transient reversals during shocks. Runs decelerate to full stop by sequentially deactivating *subsets of flagella* (see the SM [13]), producing a torque imbalance which gradually increases track curvature [Fig. 3(f)]. Two disparate timescales are evidenced: an ultrafast, millisecond timescale for bifurcations to or from shocks, and a slower one for entry into stop states. The former is reminiscent of neuronal spiking, while the latter is akin to decay of leakage currents. For the first two sequences [Fig. 3(g)], the mean is well fit to a sharply peaked Gaussian (*σ* = 8.6 and 11.6 ms, respectively), whereas run⇀stop conversions follow a switchlike tanh profile with relaxation time *τ* = 640 ms. The true maximum speed reached during shocks is likely even higher since our imaging platform limits us to 2D projections of the motion.

This timescale separation is apparent in the stop gait, in which a cell can remain for minutes. By contrast, swimming restarts in tens of milliseconds (see the SM [13]). Surprisingly, negligible cell body motion with subpixel variance in centroid displacement [Fig. 4(a); *σ*_*δC*_ = 0.0253 *μ*m] is coupled with significant flagellar activity [*O*(1) *μ*m fluctuations], and even small-amplitude oscillations [Figs. 4(b) and 4(c)]. This novel mode may be related to hyperoscillations in reactivated sperm flagella resulting from oscillations of individual dyneins [24]. At onset of stop⇀shock transitions, the emergence of limit-cycle beat oscillations is Hopf-like, occurring simultaneously in all eight flagella.

Excitability is further evidenced by an acute mechano-sensitivity, wherein shocks are induced by external stimuli, even contact with one flagellum (see the SM [13]). These stimulated shocks are identical to spontaneous shocks. Figure 5(a) shows a moving cell colliding with one at rest: contact is made multiple times but a shock is only triggered in cell 2 by a sufficient perturbation. The threshold contact force *F* = 3*EIδ / L*^3^ is estimated from the tip deflection *δ*. For a nonbeating flagellum with bending rigidity *EI* = 840 pN *μ*m^2^ [25], we have no shock when *F* ≲ 3.0 pN, but shock when *F* ≳ 6.6 pN. For multiple two-cell collisions, we measured a *O*(10) ms signal transduction from the distal point of contact to flagellar response. Thus, shocks not only effect swimming reorientations [26] but also enable ultrafast escape from predators or obstacles upon direct contact. Physiologically, this may be related to the escape responses of *Chlamydomonas* and *Spermatozopsis*, which last much longer (0.2–1.0 s) and do not occur spontaneously, requiring instead strong light or mechanical triggers [27,28].

In summary, *P. octopus* is a microswimmer capable of robust behavioral stereotypy and responsiveness in the absence of neuronal control of the kind pertaining to animal models [29,30]. Its run-stop-shock motility is a significant departure from all known strategies, such as the two-state *E. coli* run and tumble [4] and its sister eukaryotic version in *C. reinhardtii* [31–33], and different still from the run-reverse-flick motility of *Vibrio* [5,34]. Instead, gait switching in *P. octopus* solicits total conversion of beating along the flagellar axoneme proper (Fig. 2a), in which runs, shocks, and stops are coincident with the three major modes of eukaryotic flagella (*ciliary, flagellar*, and *quiescent*) [18]. This contrasts with classical gait-switching mechanisms reliant on a basal rotor or flagellar hook (as in bacteria), or on modulation of flagellar synchrony (as in *C. reinhardtii*), making *P. octopus* ideally suited for examining bifurcations between different dynamical states of the *same* organelle.

Ascribing the motility pattern to a tripartite repertoire, we shed new light on the physiology of gait control in flagellates, revealing its strongly nonequilibrium character. The measured breaking of detailed balance exposes an inherent temporal irreversibility in the control mechanism, adding further complexity to the need to enact time-irreversible beat patterns to overcome Stokes reversibility [35], while consuming chemomechanical energy. We showed that each run⇀stop⇀shock cycle elicits timescales separated by 2 orders of magnitude, corresponding to rapid activation (forward reaction) but slow deactivation (backward reaction). Our analyses suggest that active motility resides at criticality, through the observation that quiescent flagella exhibit robust small-amplitude oscillations bifurcating to full-amplitude beating when induced by noise or weak mechanical forcing. Each flagellum, operating far from equilibrium, executes highly nonlinear responses and large phase-space excursions [Fig. 2(a)]. These results have significant implications for understanding beat emergence [36,37] and motor coordination in eukaryotic cilia and flagella [38–40].

Criticality and excitability are hallmarks of nonequilibrium activity, which may promote biological sensitivity (cf. chemotaxis [41], hair cells of the inner ear [42]). *P. octopus* appears to be more reactive to noise and mechanical perturbations than other species such as *C. reinhardtii* [43–45]. For such microswimmers, optimizing for motility does not equate to enhanced sensing: the shock and stop gaits clearly contribute minimally to motility but create an added complexity which may be key to effecting heightened sensitivity and rapid responses to transient signals. In *P. octopus* this may have resulted from adaptation to a unique benthic habitat in which rapid signal transduction is critical for avoiding physical obstacles (e.g., sand grains) or predation. In more advanced phyla, cilia and flagella continue to fulfill key sensory and motile functions, switching between neurally controlled oscillatory or nonoscillatory states in ctenophores [46], and generating nodal flows for embryonic symmetry breaking [47]. Thus, in this little-known, billion-year-old unicellular marine alga, we may have found an evolutionary precedent for these highly evolved and conserved functionalities.

## Supplementary Material

SI

## Figures and Tables

**FIG. 1:**
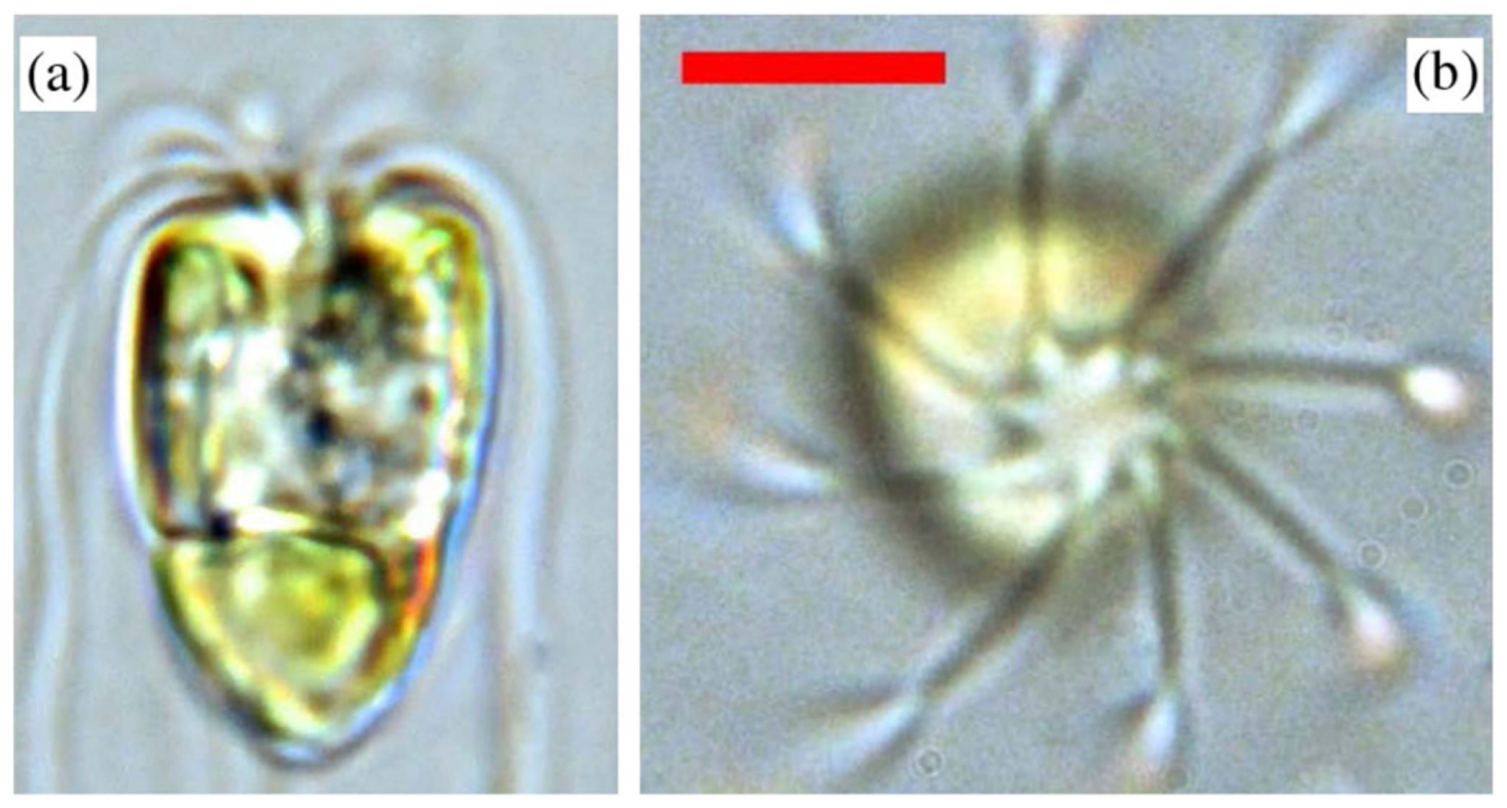
(a) Side and (b) top views of *Pyramimonas octopus* (flagella spiraling clockwise when viewed from above). The eye-spot is visible as a conspicuous orange organelle. (Scale bar, 5 *μ*m).

**FIG. 2:**
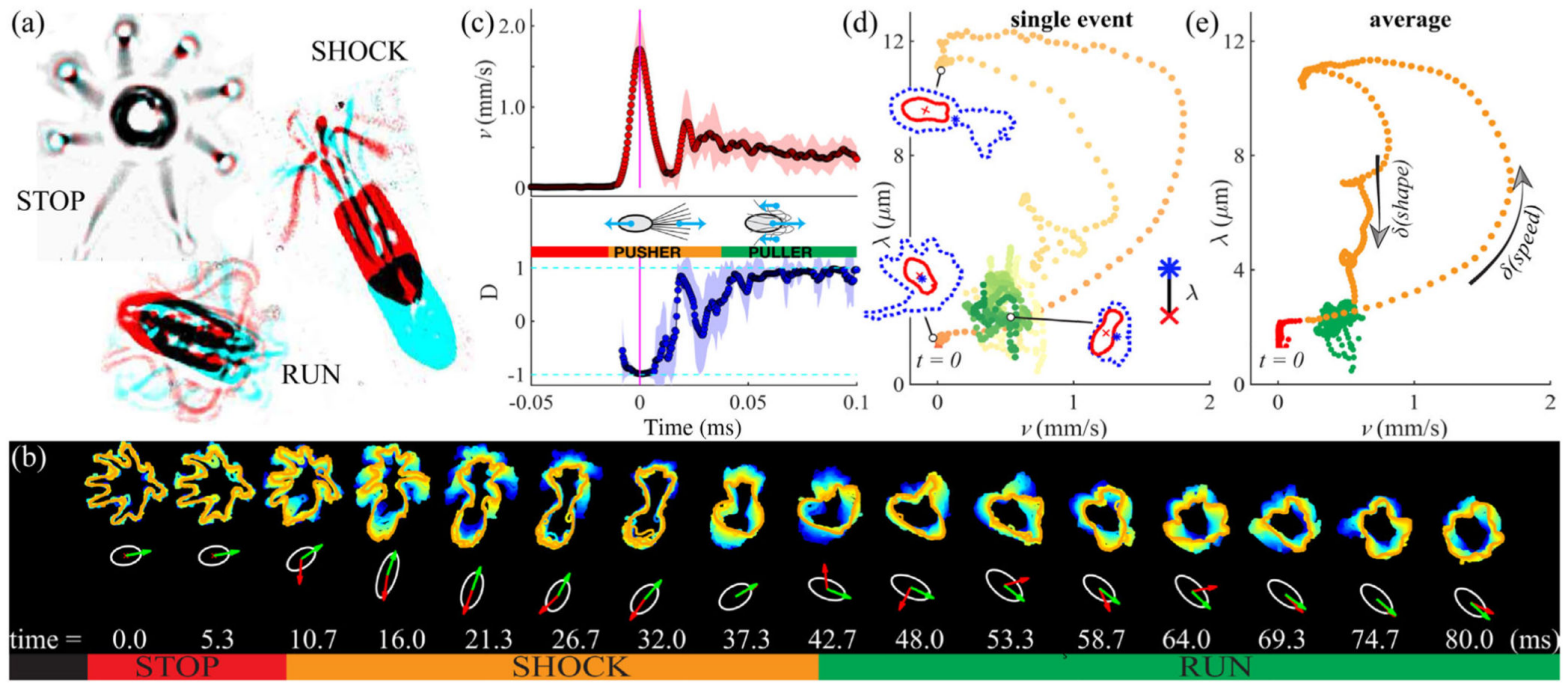
(a) Three gaits of *P. octopus*: pairs of video frames showing the cell at an initial (red) and a later time (cyan), separated, respectively, by 100, 10, 5 ms for stop, run, shock, are superimposed. (b) Dynamically changing flagellar waveforms produce cell reorientation. Here, traced flagellar envelopes are displayed on coarse (10 ms) and fine (5 ms) timescales. (White ellipses, cell body; green and red arrows, cell orientation **ê**_*R*_ and swimming direction $\hat{v}$.) (c) Transition from stop to run occurs via a shock, with rapid changes in speed *v* and alignment *D* [“pusher” to “puller” transition, shaded region = 1 standard deviation (std)]. (d), (e) Stop-shock-run sequences are plotted in speed-shape space for a single cell and for a multievent average from different individuals, and they are color coded by time. Sample cell and flagellar boundaries in (d) correspond to instants *t* = 33, 79, and 211 ms.

**FIG. 3:**
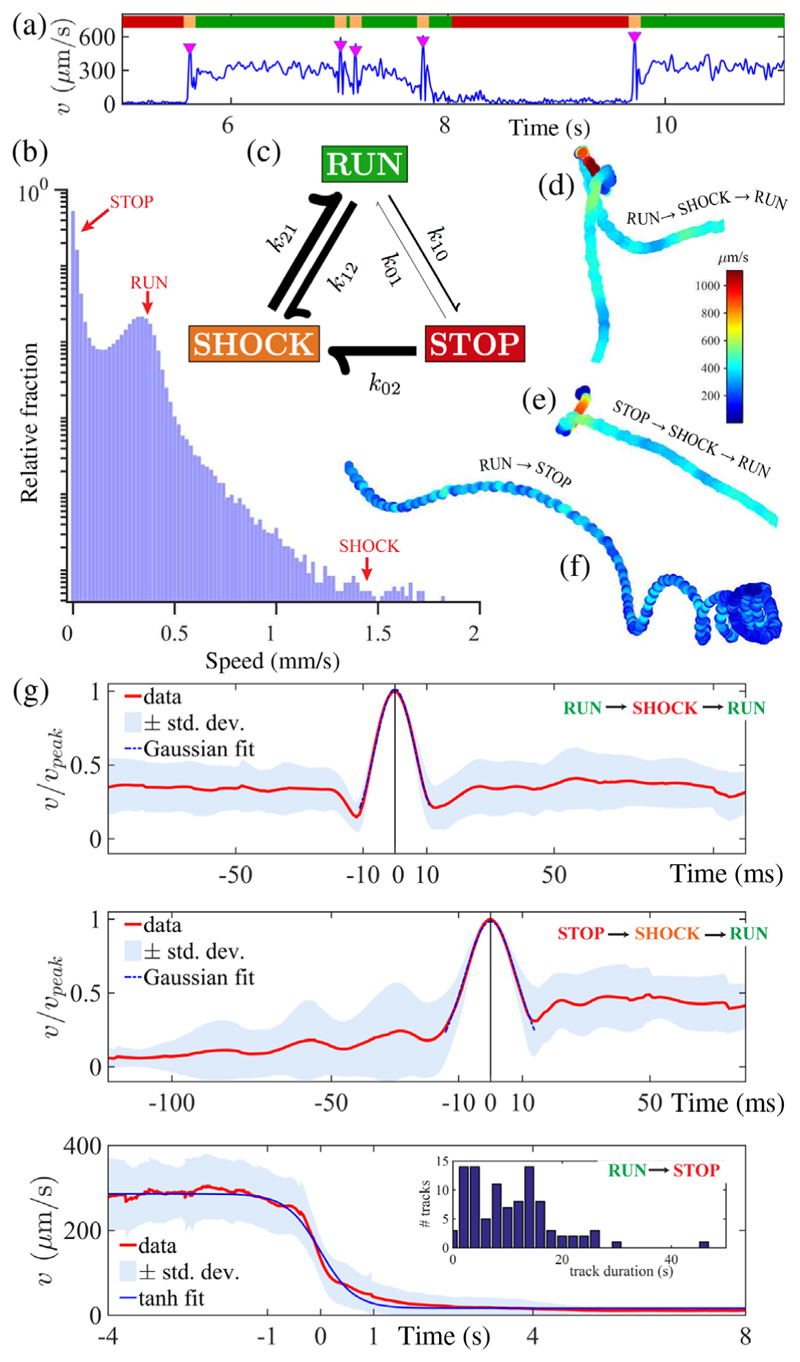
(a) Single-cell motility is partitioned by instantaneous speed *v*(*t*) into three states (0, stop; 1, run; 2, shock). Shocks are denoted by downward triangles. (b) Probability density distribution of speeds (log scale) reveal dwell times in each state. (c) Permissible gait transitions are indicated by arrows (weighted by rates *k*_*ij*_). (d)–(f) Sample trajectories for characteristic transition sequences. (g) Superimposed and averaged swimming speeds exhibit pulselike maxima during shocks, but much slower decay during run⇀stop transitions. (Inset) Histogram of track durations.

**FIG. 4:**
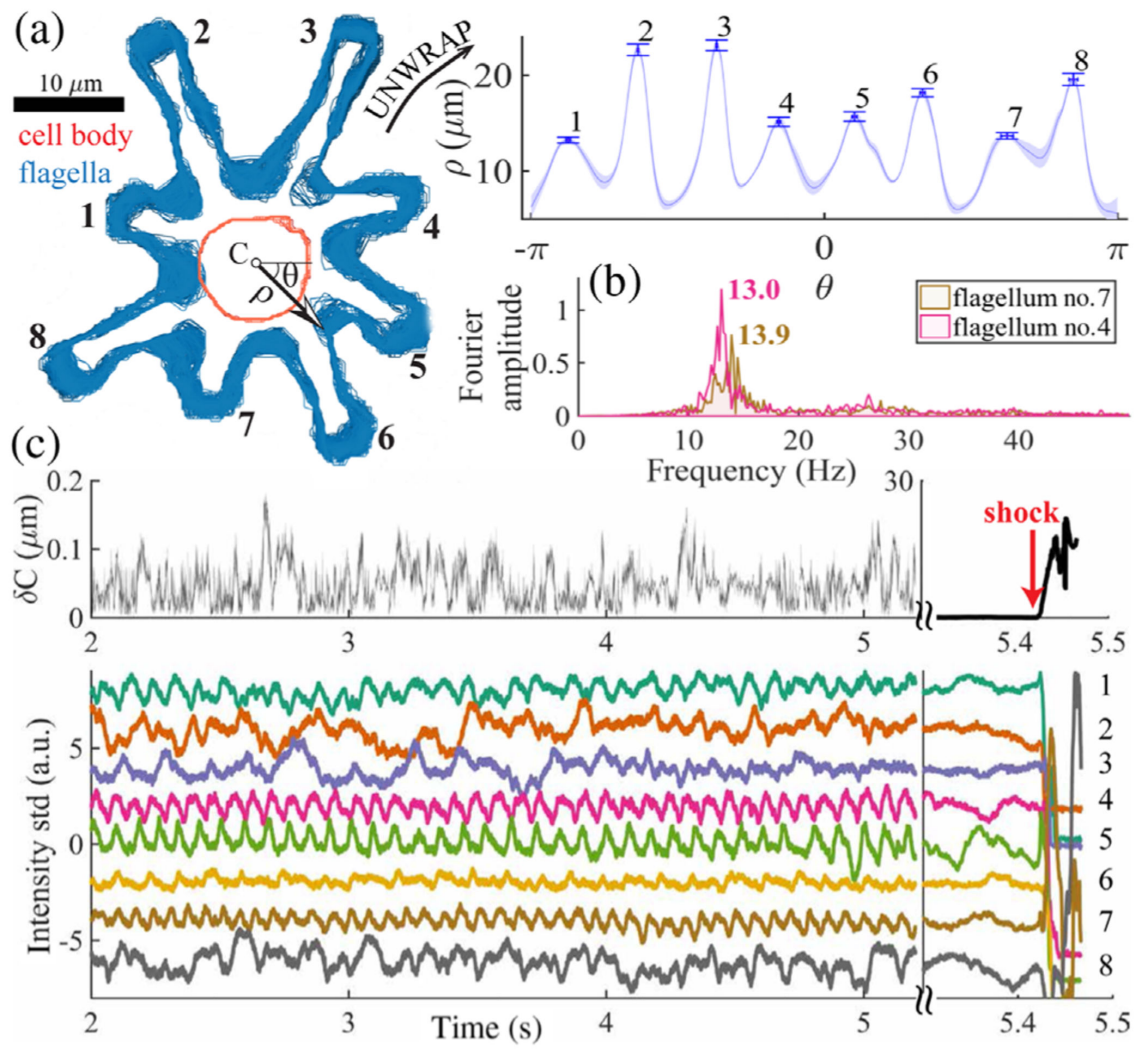
The stop gait. (a) Cell and flagellar boundaries in successive frames are superposed. (Inset) The polarly unwrapped flagella envelope exhibits micron fluctuations (see error bars; shading is one std), while (b) individual flagella display robust oscillations. (c) Cell centroid fluctuations are subpixel and random, yet flagella tips oscillate. All eight flagella bifurcate simultaneously to full-amplitude beating (shock).

**FIG. 5:**
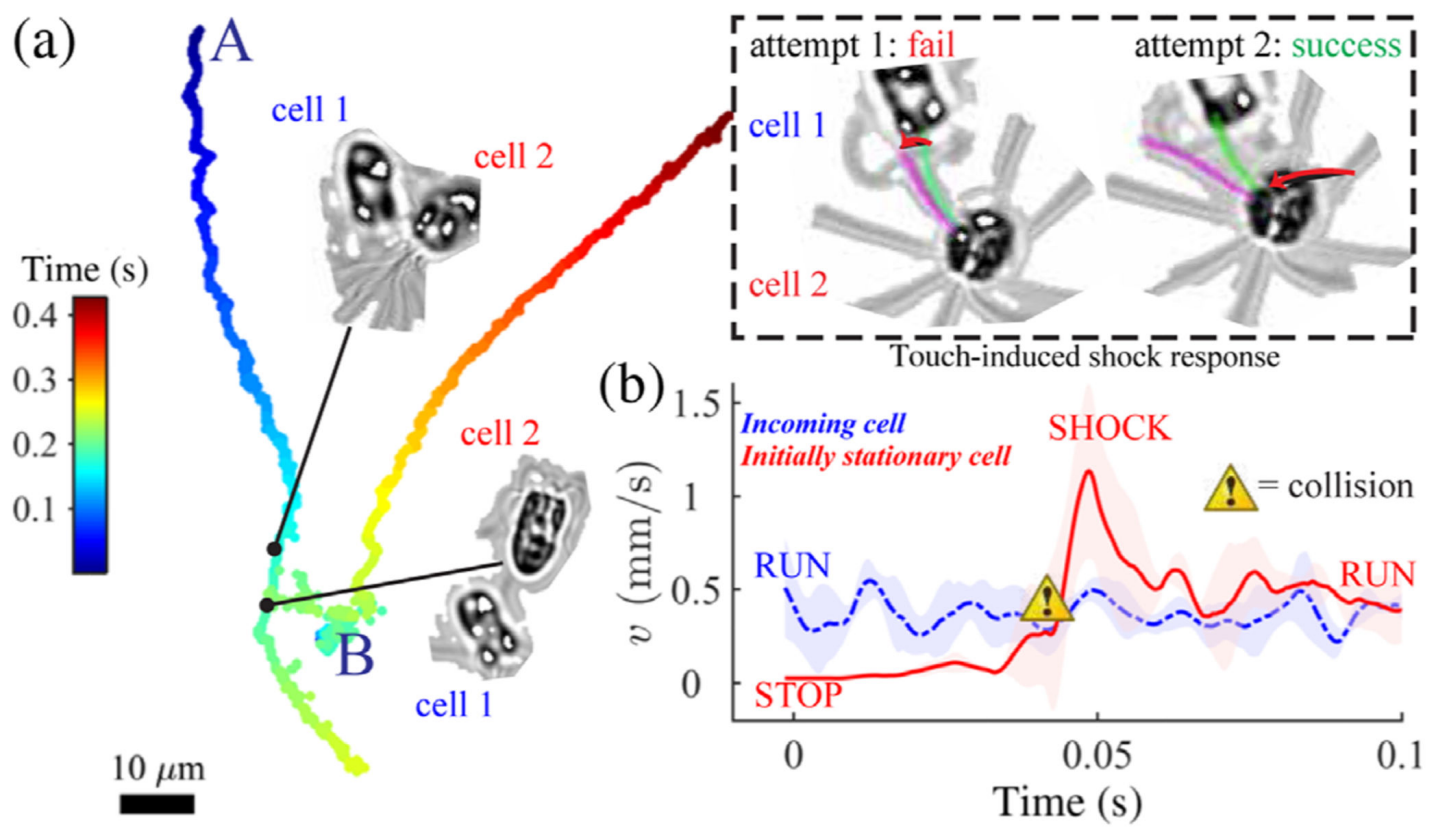
(a) Flagellar mechanosensitivity. (Inset) Mechanical contact with one flagellum is sufficient to trigger a shock given enough forcing. (b) Sequence of changes in swimming speed averaged over four sample cell-cell collisions—in each case, between a moving cell and a stationary cell.
