# Supplementary material for: Time Irreversibility and Criticality in the Motility of a Flagellate Microorganism
Source: Phys Rev Lett. Author manuscript; Available in PMC 2024 Jun 10. (PMC7616082; doi:10.1103/PhysRevLett.121.058103)
Supplement: SI [file EMS196591-supplement-SI.pdf]

Time-irreversibility and criticality in the motility of a flagellate microorganism
Supplemental Material

Kirsty Y. Wan[1,2] and Raymond E. Goldstein[1]

1. Department of Applied Mathematics and Theoretical Physics, Centre for Mathematical Sciences, University of Cambridge, Wilberforce Road, Cambridge CB3 0WA, United Kingdom
2. Living Systems Institute, University of Exeter, Stocker Road, Exeter EX4 4QD, United Kingdom

In the following, we give further details of some of the experimental, imaging, analysis and modelling employed in the main text.

1. Overview of experimental methods

Cell cultures were obtained from the Scandanavian Collection of Algae and Protozoa (SCCAP K-0001, *P. octopus* Moestrup et Aa. Kristiansen 1987), and grown in Guillard's F/2 medium under controlled illumination (14:10 day/night diurnal cycle, at 22°C). Cells are oblong or rectangular in aspect (Fig. 1), with length (17.05 ± 1.74 μm) and width (9.05 ± 1.23 μm). In their vegetative state cells have 8 flagella, each of length comparable to the longitudinal dimension of the cell body, which emerge radially from an apical grove Imaging was conducted under white light illumination on an inverted microscope (Nikon Eclipse TE2000-U) and high-speed recordings made at up to 3000 fps (Phantom v311, Vision Research). Organisms were harvested during exponential growth (at $10^4 - 10^5$ cells/cm^3), and $50 - 150$ μl of suspension were pipetted gently into shallow quasi-2D chambers (top + bottom: glass, side: Frame-Seal slide chambers – BIO-RAD) for imaging and precision cell and flagella tracking via custom Matlab algorithms and ImageJ extensions – see Supplemental Materials (SM). We ensured cell viability by minimizing environmental stress responses: acclimating cells prior to observation, and limiting continuous light exposure to $\lesssim 15$ minutes.

2. A pusher-puller transition

For free-swimming microorganisms such as algae or bacteria, the motion of the cell is tightly coupled to the motion of the flagella [2, 3]. Our results show that sharp reorientations in swimming trajectories in *P. octopus* are elicited by dramatic conversion of flagellar beating waveforms. Cells have a well-defined length-width aspect ratio β which we determined from a large sample population $\mathcal{O}(100)$ of cells to be $\beta = 1.9 \pm 0.2$.

Importantly $\beta \neq 1$ which allows us to distinguish the longitudinal and transverse directions of a organism moving in the focal plane. The anterior and posterior poles are defined by the user on frame t_0 of each movie, but tracked automatically thereafter.

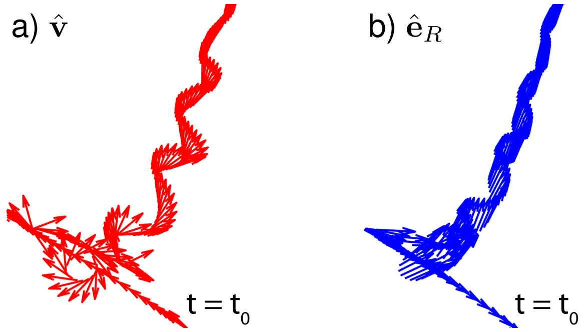

FIG. S1. Cell orientation versus direction of swimming: the puller-pusher dichotomy.

For a given trajectory, we obtain 2D coordinates for the anterior $\mathbf{A}_i$(t), posterior $\mathbf{P}_i(t)$, and centroid of the cell $\mathbf{C}_i(t)$, at discrete times indexed by $t_0, t_1, t_2, \cdots$, $(\Delta t = t_{j+1} - t_j)$. From this we take the instantaneous swimming direction $\hat{\mathbf{v}}$ and the instantaneous orientation $\hat{\mathbf{e}}_R$ to be

$$\mathbf{v}(t_j) = \frac{\mathbf{C}(t_{j+1}) - \mathbf{C}(t_{j-1})}{2\Delta t} \quad (1)$$

$$\mathbf{e}_R(t_j) = \mathbf{A}(t_j) - \mathbf{P}(t_j) \quad (2)$$

and define $\hat{\mathbf{v}} = \mathbf{v}/||\mathbf{v}||$, $\hat{\mathbf{e}}_R = \mathbf{e}_R/||\mathbf{e}_R||$.

In Figure S1 the two quantities are plotted following the same trajectory, starting at $t = t_0$, which shows a cell switching from a backward swimming, pusher-like shock gait to a forward, puller-like run gait. The recovery from a flagellar to ciliary beat is concomitant with a continuous modulation of the swimming direction. At this magnification, we see that $\hat{\mathbf{v}}$ is everywhere tangent to a helical run trajectory (due to self-rotation).

3. Tracing moving boundaries

At sufficiently high magnification, it is possible to distinguish the cell body from the flagella bundle. For this, we take advantage of the differences in image intensity

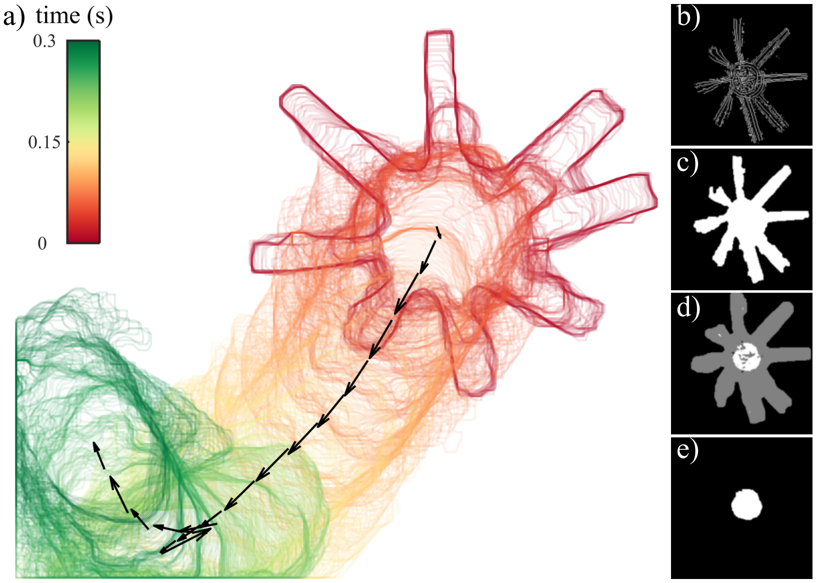

FIG. S2. a) The flagellar envelope is traced for a cell starting from a stopped state. Arrows follow the direction of motion of the cell body. The processing algorithm used to delineate the inner and outer cell boundaries, used for computing λ (Fig. 2c, main text), is summarized in b-e).

features between the cell body and flagella. The algorithm was written in MATLAB with the aid of the Image Processing Toolbox (version 9.5). Fig. S2 shows the morphing outer boundary of the organism (exterior of the flagella) as it transitions from a stopped state (red), undergoes a shock response (amber), before moving off in a new direction (green). The exterior boundary was detected from the raw intensity image using the Sobel method (b) and by morphological dilation and erosion of the edge-like features, as required. To further detect the inner boundary (cell body only), we restrict to and quantize the sub-image bounded by this external border (d), into the darker central region and the surrounding pixels that are not part of the background. We estimate that the edge-detection algorithm introduces an error of $\lesssim 0.5\mu$m in the localization of the true boundary. Finally, the image is binarized to obtain (e).

4. Transition Probabilities between states

The instantaneous swimming speed v is an efficient means of segregating the observed dynamics into the three states of interest. The true speed may differ slightly from this as we are observing only a two-dimensional projection of the fully three-dimensional swimming dynamics, but this difference is small and has little impact on the discretization. A combination of signal processing criteria (filtering by minimum peak height, minimum peak-to-peak separation etc) was used to discretize v into the three states: $0 =$ stop, $1 =$ run, $2 =$ shock.

Model formulation In order to analyse the likelihood of gait-switching, we model the underlying stochastic process generating the empirical data as a continuous time, discrete space Markov chain for states $X(t) \in \{0, 1, 2\}$. In reality X is measured at a succession of times discretized by imaging frame-rate, which we assume to be sufficient to provide the necessary temporal resolution. The Markov assumption is motivated by the data; in Fig. S3 we plot the interval distributions between successive events. In the case of runs and stops, the waiting times between runs (respectively stops) are seen to be approximately exponentially distributed. In the case of shocks the waiting time distributions have a very rapid decay due to the very fast nature of these reactions. The latter timescale is at the limit of our imaging resolution and therefore we do not preclude the possibility that the occurrence of shocks may not be memoryless. Indeed if future experiments could prove that interval distributions for shocks are not memoryless, this would be additional evidence for non-equilibrium, excitable gait-control rem-

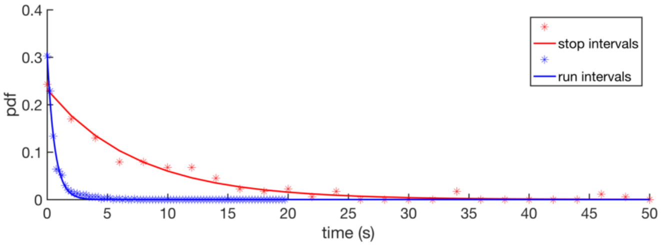

FIG. S3. Waiting time distributions between successive runs and successive stop events.

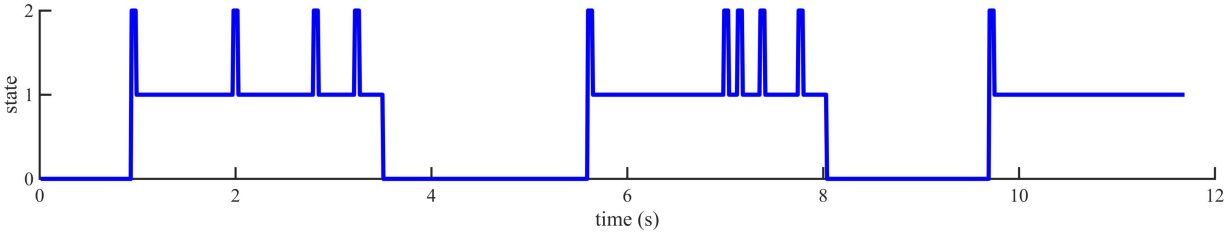

FIG. S4. A sample track showing discrete transitions between three states (0 = stop, 1 = run, 2 = shock), is obtained by discretizing the instantaneous cell swimming speed.

iniscent of neuronal firing.

We invoke the Markov model here as a means of measuring transition rates between states and their relative likelihoods. Multiple tracks are sampled to obtain longitudinal data in the form $\{X_{mn}, m = 1, 2, \ldots, N, n = 1, 2, \cdots, m_N\}$, for a total of N-tracks corresponding to different cells, and in which each track m is observed for m_N frames. The transition intensity is given by

$$q_{ij}(s,t) = \lim_{s \to t} \frac{\mathcal{P}(X(t) = j | X(s) = i)}{t - s}, \tag{3}$$

which represents the risk of q_{ij} of moving into state j at time t starting from state i at time s. We shall assume the Markov property, that is for $t_0 < t_1 < \cdots < t_n$,

$$\begin{aligned} &\mathcal{P}(X(t_n) = i_n | X(t_0) = i_0, \cdots, X(t_{n-1}) = i_{n-1}) \\ &= \mathcal{P}(X(t_n) = i_n | X(t_{n-1} = i_{n-1}), \end{aligned} \tag{4}$$

and that the process is homogeneous: $\mathcal{P}(X(t) = j | X(s) = i) = \mathcal{P}(X(t-s) = j | X(0) = i) =: p_{ij}(t - s)$. The instantaneous probability distribution $P_i(t) = \mathcal{P}(X(t) = i))$ $(\sum_i P_i(t) = 1)$ is then completely determined by the initial distribution $p_0 = P_i(0)$ and the infinitesimal transition rate matrix $Q = \{q_{ij}\}$. Off diagonal entries $q_{ij} > 0$ for $i \neq j$ are are given by eqn (4), while diagonal entries

$$q_{ii} = -\sum_{j \neq i} q_{ij}.$$

The transition matrix $P = \{p_{ij}\}$ satisfies the Chapman-Kolomogov equations $p_{ij}(s,t) = \sum_k p_{ik}(s,u) p_{kj}(u,t)$, and the matrix differential equation $dP/dt = P(t)Q$ (forward equation), with solution

$$P(t) = \exp(Qt) = \sum_{k=0}^{\infty} \frac{Q^k t^k}{k!}. \tag{5}$$

Defining the sequence $\{T_n\}_{n \in \mathbb{N}}$ of jump times

$$T_{n+1} = \inf\{t \geq T_n | X(t) \neq X(T_n)\}, \tag{6}$$

then the sojourn times $S_n = T_n - T_{n-1}$ are exponentially distributed with rate $\lambda = -q_{ii}$, i.e.

$$\mathcal{P}(S_n \leq t) = 1 - \exp(q_{ii} t),$$

and moreover this new state is $j \neq i$ with probability

$$\lim_{h \to 0} \frac{\mathcal{P}(X(t+h) = j | X(t) = i)}{\mathcal{P}(X(t+h) \neq i | X(t) = i)} = -\frac{q_{ij}}{q_{ii}}. \tag{7}$$

Then the stochastic matrix given by:

$$\tilde{p}_{ij} = \begin{cases} 1 & (q_i = 0; j = i) \\ 0 & (q_i = 0; j \neq i) \\ -q_{ij}/q_{ii} & (q_i \neq 0; j \neq i) \\ 0 & (q_i \neq 0; j = i) \end{cases} \tag{8}$$

defines a transition matrix for an *embedded Markov chain*, where the $\tilde{p}_{ij}$ are the probabilities that given a transition occurs, the state moves from i to j. The embedded chain has no self-transitions.

Recall that a finite-state irreducible Markov chain is positive recurrent, so in this case we expect a unique stationary distribution π to exist, and to satisfy:

$$\lim_{t \to \infty} P(t) = \mathbb{1}\pi.$$

The process is *time-reversible* iff in detailed balance

$$\pi_i q_{ij} = \pi_j q_{ji}$$

Results We obtained a total of $N = 233$ tracks each containing at least one transition event, with mean track duration 11.5 s and maximum track duration 78.2 s. The data is reshaped so that there is only one row per transition, corresponding to observations for a given cell. Next we sub-sample so that only jump-times are retained $\{X_{mn_k} : t_{n_k} \textit{corresponding to jump times } T_k^m\}$ in the sense of eqn (6). A total of 1377 pairwise transitions were observed, with the following frequencies

	stop	*run*	*shock*
stop	0	0.005	0.070
run	0.085	0	0.317
shock	0	0.523	0

The data was fitted to the above Markov state model using the R-software package msm [1] to obtain maximum likelihood estimates for unknown parameters. Let $\{T_i\}$ be the total time the process is observed in each state, N_i the total number of observed transitions from state i,

and N_{ij} the number of transitions from i to j. We can use eqn (7) to initialize the Q-matrix, where N_{ij}/N_i is an estimate for $-q_{ij}/q_{ii}$ and T_i/N_i to estimate the mean waiting time in state i (expected to be $-1/q_{ii}$). Thus, $\hat{q}_{ij} = N_{ij}/T_i$.

The following Q-matrix was obtained (together with 95% confidence intervals)

$$\begin{array}{c} \\ stop \\ run \\ shock \end{array} \begin{array}{c} \begin{array}{ccc} \qquad stop \qquad & \qquad run \qquad & \qquad shock \end{array} \\ \begin{bmatrix} -0.13166\ (-0.159709, -0.10854) & 0.00767\ (0.003446, 0.01707) & 0.12399\ (0.101617, 0.15129) \\ 0.28075\ (0.234222, 0.33652) & -1.32937\ (-1.444803, -1.22315) & 1.04862\ (0.954768, 1.15169) \\ 0 & 19.76936\ (18.376811, 21.26743) & -19.76936\ (-21.267428, -18.37681) \end{bmatrix} \end{array} \tag{9}$$

We can also compute the transition matrix $P(t)$, which estimates the transition probabilities at different times.

$$P(0.01) = \begin{array}{c} \\ stop \\ run \\ shock \end{array} \begin{array}{c} \begin{array}{ccc} stop & run & shock \end{array} \\ \begin{bmatrix} 0.9987 & 0.0002 & 0.0011 \\ 0.0028 & 0.9878 & 0.0095 \\ 0.0003 & 0.1782 & 0.8215 \end{bmatrix} \end{array} \tag{10}$$

$$P(0.1) = \begin{array}{c} \\ stop \\ run \\ shock \end{array} \begin{array}{c} \begin{array}{ccc} stop & run & shock \end{array} \\ \begin{bmatrix} 0.9870 & 0.0074 & 0.0055 \\ 0.0267 & 0.9299 & 0.0434 \\ 0.0152 & 0.8163 & 0.1685 \end{bmatrix} \end{array} \tag{11}$$

$$P(1) = \begin{array}{c} \\ stop \\ run \\ shock \end{array} \begin{array}{c} \begin{array}{ccc} stop & run & shock \end{array} \\ \begin{bmatrix} 0.8902 & 0.0992 & 0.0106 \\ 0.2201 & 0.7389 & 0.0410 \\ 0.2109 & 0.7477 & 0.0414 \end{bmatrix} \end{array} \tag{12}$$

where time is measured in units of seconds, and stochastic matrices are truncated to 4 decimal places. The above has interesting interpretations, for instance the shock state is confirmed to be the most transient, since the probability of arriving from a shock state to another shock state dropping from 0.8215 at time $t = 0.01$ s down to 0.1685 at $t = 0.1$ s.

As $t \to \infty$, the rows of P converges to:

$$\pi(stop, run, shock) = (0.6666, 0.3126, 0.0208)\,.$$

The MLEs for diagonal entries $\hat{q}_{ii}$, by the Markov assumption, give estimates (together with standard errors) for the mean waiting time in state i:

$$\begin{aligned} &\{E(T_i) = -\,1/\hat{q}_{ii}\}(stop, run, shock) \\ &\qquad = (7.60 \pm 0.75, 0.75 \pm 0.03, 0.05 \pm 0.002)\,, \end{aligned}$$

Futhermore, the transition matrix for the embedded Markov process [eqn (8)] is given by

$$\tilde{q}_{ij} = \begin{array}{c} \\ stop \\ run \\ shock \end{array} \begin{array}{c} \begin{array}{ccc} stop & run & shock \end{array} \\ \begin{bmatrix} 0 & 0.0582 & 0.9418 \\ 0.2112 & 0 & 0.7888 \\ 0 & 1.0000 & 0 \end{bmatrix} \end{array}$$

Finally, we can estimate the relative time spent in each state within a certain time window (t_0, t_1). This is different from the expected waiting times – which is only for single stays. In the long time limit as $t_1 - t_0 \to \infty$ we recover the stationary distribution π_i. For

$$\frac{1}{t_1 - t_0} \int_0^{10} P(t)\, dt = (stop : 0.71, run : 0.27, shock : 0.02)\,.$$

Our multistate Markov model provides a convenient, non-invasive, image-based method for computing gait-transition parameters, and how these depend on the cell's extrinsic environment. In a separate manuscript, currently under preparation, we will evaluate and detail the dependence of transition probabilities (as a measure of departure from this non-equilibrium steady-state), on purposeful environmental stimuli.

5. An(other) example of a single-cell trajectory

A cell experiencing multiple shocks in quick succession makes several sharp turns (Fig. S5). Given a $2D$ set of trajectory coordinates $r(t) = (x_1(t), x_2(t))$ obtained from tracking of cell centroids, we measure instantaneously speeds $v = ||\mathbf{v}(t)||$ as before (recall Eqn. 1). Writing $\mathbf{v}(t) = v(\cos(\theta), \sin(\theta))$, we define the angular speed $|\omega|$ (where $|.|$ denotes absolute value):

$$\omega(t) = \frac{1}{2\Delta t}(\theta(t + \Delta t) - \theta(t - \Delta t))\,.$$

However, the orientation variable $\theta(t)$ is especially sensitive to noise, and derivative computations become problematic at high frame rates.

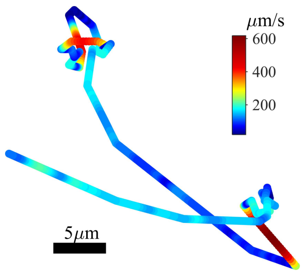

FIG. S5. Cumulative effect of shocks on a trajectory.

We resolve this problem by obtaining a track simplification via a recursive Ramer-Douglas-Peucker algorithm [4] with a relative tolerance of $0.5/\gamma$, where γ is the calibration for the number of μms per pixel on the image frame. Briefly, the algorithm recursively removes points that are lie within the given tolerance of the line defined by the end points obtained from the previous iteration. The output polygonal line is then a simplification of the original trajectory, with the advantage that sharp discontinuities are usually preserved. ω can then be determined from the simplified track (Fig. S6).

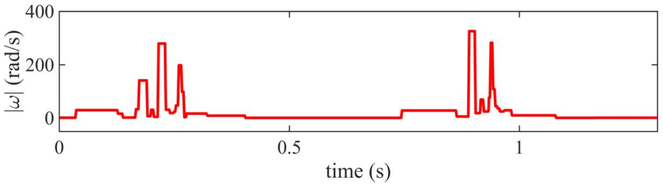

FIG. S6. Angular speed during shocks can reach several hundred radians per second.

6. Velocity pulse alignment

A few additional comments are in order regarding the signal alignment procedure used in Fig. 3g. In each case, the alignment must be determined relative to a well-defined feature of interest.

The first two sequences (run ⇀ shock ⇀ run or stop ⇀ shock ⇀ run) contain shock events, which correspond to highly reproducible pulse-like signatures in the timeseries of speeds, which provide a natural reference point – all signals are time-shifted so that the origin is at these local minima. However, we should note that cells can undergo significant out-of-plane reorientations during shocks so that the maximum speed reached is highly variable. For instance the centroid motion of a cell that reverses perpendicularly to the focal plane will not be detected in a 2D projection. Future work could seek to improve tracking fidelity by extend the imaging to 3D.

In Fig. 3g, we focused on determining a common *timescale* for shocks by rescaling the ith sequence by v^i_{max}, that is, $t = 0$ where $v^i_{max} = \max_{t_{min} \leq t \leq t_{max}} v^i(t)$. The most accurate estimate for the maximum speeds reached during shocks was calculated to be $1,712 \pm 392$m/s (see Fig. 2b), accounting for only those individuals for whom shocks occurred in the focal plane.

However there are no such peaks in run ⇀ stop transitions. In this case we make use of the derivative of the velocity instead, obtained by first filtering the signal with a lowpass filter. The decay response from run speed to full stop exhibits a tanh-like or hyperbolic profile, centred about the time point where the derivative is most negative (Fig. S7).

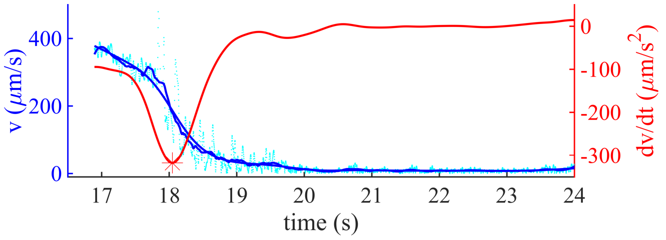

FIG. S7. Alignment of velocities for run ⇀ stop transitions using local maxima in *acceleration*. Peak *de*celeration occurs at the time indicated by the asterisk.

7. Flagellar activation versus flagellar deactivation

In the main text, we have discussed the separation in timescales between a very fast activation and a much slower deactivation. Here, we show further how activation generally involves simultaneous bifurcation to full-amplitude oscillations but yet in the reverse process the flagella do not stop beating at the same time.

Partial deactivation. A cell undergoing a run ⇀ stop transition requires several seconds to slow down to rest from full speed. The continuous convergence of $\theta(t)$ to a fixed orientation angle is shown on Fig. S8. The trajectory loops several times before terminating, as a result of propulsive forces produces by a subset of flagella that continues to beat while the remainder have already stopped.

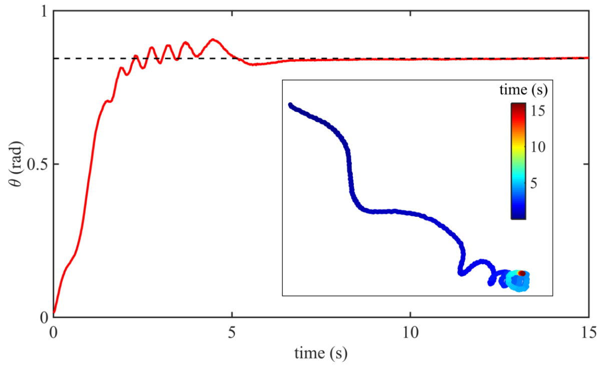

FIG. S8. Evolution of orientation angle over time, with corresponding trajectory (inset).

Simultaneous activation. In Fig. S9 we follow the exterior boundary traced by the flagella during a stop ⇀ shock transition. Within 20 ms, the outline has morphed from the red outline to the green. In particular, by identifying peaks corresponding to the 8 flagella (from unwrapped polar angle), we can obtain the trajectories of each of the flagella *tips* during this process (dark to light indicates increasing time coordinate).

Slow small-amplitude oscillations. Finally, recall that the eight flagella shown in Fig. 4, to varying degrees, exhibit oscillations. To investigate if there is *global* period-

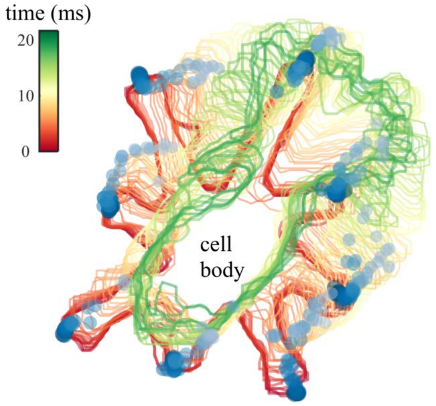

FIG. S9. Simultaneous activation of all flagella at shock onset.

icity, Instead of measuring interflagellar cross-correlation functions, we consider the total area spanned by the flagellar envelope (487 ± 9.88 μm), which fluctuates over time. The existence of a $12 \sim 14$ Hz oscillation is evident from the auto-correlation function $C_{area}(\tau) = \langle X(t)X(t+\tau)\rangle_\tau$ (Fig. S10).

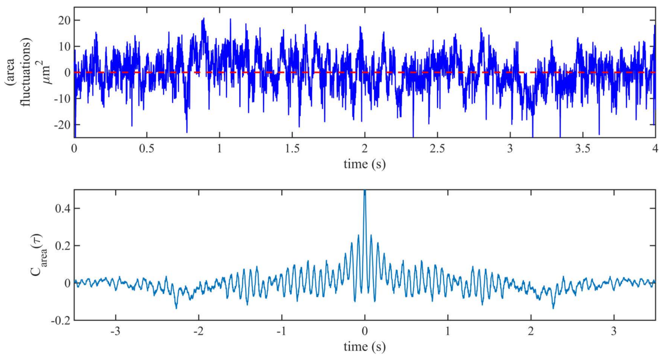

FIG. S10. Area fluctuations (a) and its auto-correlation function (b) for a cell observed during the stop state.

8. Captions for supplemental movies

MovieSM1 threesequences Examples of the three primary sequences discussed in the text, namely run ⇀ shock ⇀ run, stop ⇀ shock ⇀ run, and run ⇀ stop.

MovieSM2 quiverandshock A cell is observed during the stop state. Despite no cell body motion, the flagella continue to 'quiver'. Remarkably, these fluctuations or vibrations are not random, but instead exhibit very small-amplitude, slow oscillations.

MovieSM3 sixcollisions We show 6 different instances in which mechanical contact by flagella stimualtes shock reactions in initially stationary *P. octopus* cells. The first five are one-on-one collisions between two individuals, while the final example shows a succession of collisions involving 4 individuals.

[1] C.H. Jackson, J. Stat. Software **38**, 1 (2011).
[2] G.B. Witman, Trends Cell Biol. **3**, 403 (1993).
[3] O. Moestrup, Protoplasma **148**, 41 (1989).
[4] W. Schwanghart, *Line Simplification* version 1.4, MATLAB Central File Exchange, (2010).
